# Supplementary material for: Effectiveness of nirmatrelvir/ritonavir in children and adolescents aged 12–17 years following SARS-CoV-2 Omicron infection: A target trial emulation
Source: Nat Commun. 2024 Jun 8;15:4917. doi: 10.1038/s41467-024-49235-8 (PMC11162460; doi:10.1038/s41467-024-49235-8)
Supplement: Supplementary file 1 — Supplementary Information [file 41467_2024_49235_MOESM1_ESM.pdf]

Supplementary Material

Supplementary Figure 1. Boxplot of logarithm of the inverse probability of censoring weights during treatment initiation period (day 1-5) in nirmatrelvir/ritonavir and control groups

Supplementary Table 1. Summary of the study protocol of emulating a target trial using observational data

Supplementary Table 2. Definitions of secondary study outcomes

Supplementary Table 3. Demographics and clinical characteristics of nirmatrelvir/ritonavir group and control group at the end of the 5-day grace period before the inverse probability of censoring weighting (IPCW)

Supplementary Table 4. Cause of hospitalization among nirmatrelvir/ritonavir users and controls after cloning and censoring

Supplementary Table 5. Sensitivity analyses on the 28-day all-cause hospitalization outcome by (1) truncating the IPCW at the 1st and 99th percentiles to minimize the impact of extreme weights on the results and (2) extending the follow-up duration till the end of observational period

Supplementary Table 6. Cumulative incidences of secondary study outcomes at 28 days in nirmatrelvir/ritonavir and control groups

Supplementary References

Supplementary Figure 1. Boxplot of logarithm of the inverse probability of censoring weights during treatment initiation period (day 1-5) in nirmatrelvir/ritonavir and control groups

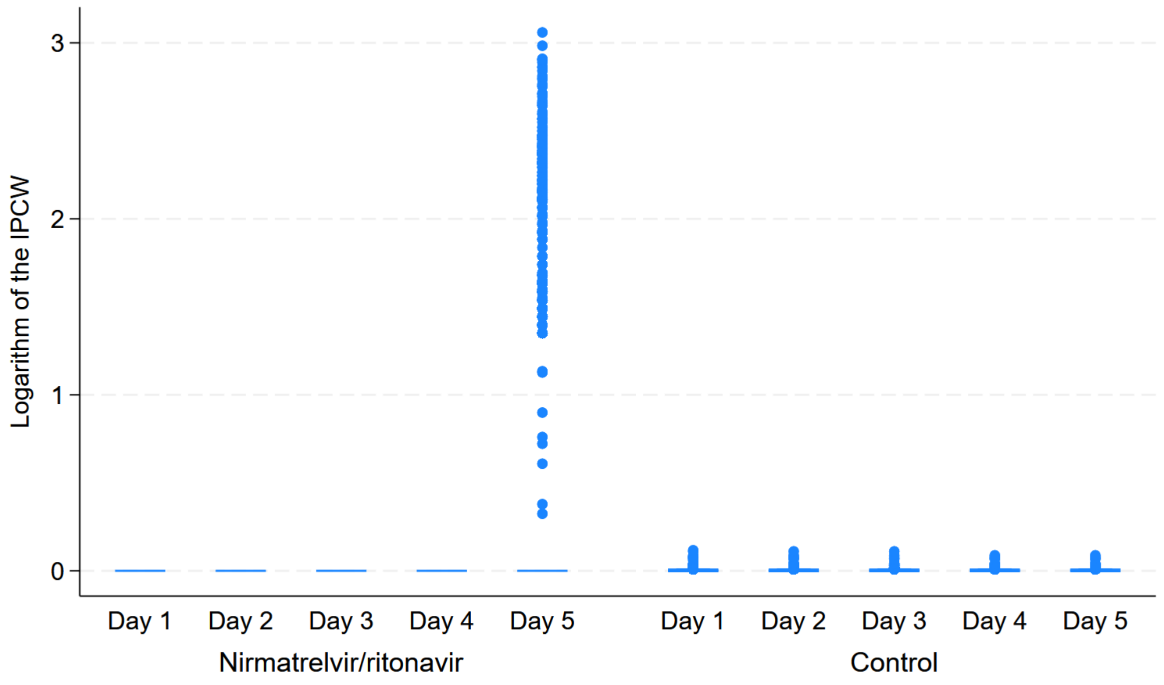

	Construction of IPCW[1,2]	
Time point	Nirmatrelvir/ritonavir	Control
Days 1-4 (during grace period)	1	1/(1-*p_treatment*)
Day 5 (end of grace period)	1/*p_treatment*	1/(1-*p_treatment*)
Days >5 (after grace period)	1	1/(1-*p_treatment*)

Notes:

Boxes show median as center line; box limits indicate 25th and 75th percentiles; whiskers extend from 25th percentile less 1.5 times inter-quartile range to 75th percentile plus 1.5 times inter-quartile range; points beyond whiskers represent outliers.

p_treatment: The probability of initiating nirmatrelvir/ritonavir treatment at each time point conditional on baseline covariates.

The weights during days 1-4 and days >5 were set to 1 in the nirmatrelvir/ritonavir group because no patients were artificially censored due to change in treatment strategy during these two periods. As there was no more informative censoring after the 5-day grace period, the probability of being censored was 0 and the daily weight was 1 for days >5 in both groups. The estimated IPCW, which was the cumulative product of daily weights since day 1, stayed constant from days >5 onwards.

Supplementary Table 1. Summary of the study protocol of emulating a target trial using observational data

Protocol component	Target trial specification	Emulation using observational data
Eligibility criteria	Aged 12-17 years, with confirmed SARS-CoV-2 infection diagnosis during the study inclusion period<br><br>Patients are excluded if:<br>• Hospitalized or dead on or before the SARS-CoV-2 infection diagnosis<br>• With severe renal impairment (estimated glomerular filtration rate [eGFR] <30 mL/min/1.73m^2, dialysis, or renal transplantation)<br>• With severe liver impairment (cirrhosis, hepatocellular carcinoma, or liver transplantation)<br>• With drug contraindications to nirmatrelvir/ritonavir<br><br>Index date is defined as that of SARS-CoV-2 infection diagnosis	Same as specification<br><br>Date of confirmed SARS-CoV-2 infection diagnosis is the date of first positive reverse transcription polymerase chain reaction or rapid antigen test result during the study inclusion period.
Treatment strategy	Initiation of nirmatrelvir/ritonavir within five days of symptom onset	Initiation of nirmatrelvir/ritonavir within five days of the index date
Assignment procedures	Patients are randomly assigned to receive nirmatrelvir/ritonavir in addition to usual care versus usual care alone.	Patients will be classified into treatment or control groups based on their drug dispensing and prescription records during the first five days (grace period) from the index date.<br><br>Random assignment of the oral antiviral treatment is assumed by the adoption of inverse probability of censoring weighting (IPCW) from the cloning method.
Outcomes	Primary outcome: 28-day all-cause mortality or all-cause hospitalization	Same as specification

	Secondary outcomes:<br>• 28-day COVID-19-specific hospitalization<br>• 28-day in-hospital disease progression (a composite outcome of in-hospital death, invasive mechanical ventilation, or admission to intensive care unit)<br>• Multisystem inflammatory syndrome in children (MIS-C)<br>• Acute liver injury<br>• Acute renal failure<br>• Acute respiratory distress syndrome	
Follow-up period	Patients are observed from the index date (time zero) to the earliest of event occurrence, date of registered death, 28 days after the index date, or the administrative end of the follow-up period (12th February 2023).	Same as specification
Causal contrasts of interest	Intention-to-treat effect<br>Per-protocol effect	Observational analogue of the per-protocol effect
Analysis plan	Intention-to-treat analysis<br>Per-protocol analysis: patients will be censored when they deviate from the respective treatment strategies (treatment or control)	Same as per-protocol analysis, adjusted for baseline confounders

Supplementary Table 2. Definitions of secondary study outcomes

Secondary outcomes	ICD-9-CM	Laboratory and other criteria
Multisystem inflammatory syndrome in children (MIS-C)	446.1	According to US Centers for Disease Control and Prevention case definition for multisystem inflammatory syndrome in children[3]
Acute liver injury	570	Satisfying at least one of the following conditions: (i) increase in alanine transaminase (ALT) was over two times the upper limit of normal (ULN); (ii) increase in aspartate transferase (AST) was over two times the ULN; (iii) increase in total bilirubin was over two times the ULN; or (iv) the international normalised ratio (INR) was over 1.5. The ULN of ALT, AST and total bilirubin were defined as 40 U-L, 40 U-L, and 19 µmol-L, respectively. (According to the Asia Pacific Association of Study of Liver consensus guidelines[4])
Acute renal failure	584.x	Satisfying at least one of the following conditions: (i) increase in serum creatinine (SCr) by 0.3 mg/dL within 48 hours; (ii) increase in SCr to 1.5 times of baseline, which was known or presumed to have occurred within the week prior (According to KDIGO Clinical Practice Guideline for Acute Kidney Injury[5])
Acute respiratory distress syndrome	518.5 518.81 518.82	PaO2-FiO2 ratio < 40 kPa

Supplementary Table 3. Demographics and clinical characteristics of nirmatrelvir/ritonavir group and control group at the end of the 5-day grace period before the inverse probability of censoring weighting (IPCW)

Characteristics	Nirmatrelvir/ritonavir users (N=345)		Controls (N=48,823)		SMD
	N / Mean	% / SD	N / Mean	% / SD	
Age, years	14.9	1.6	14.4	1.7	0.26
12-13	83	24.1%	17,068	35.0%	
14-15	123	35.7%	16,142	33.1%	0.25
16-17	139	40.3%	15,613	32.0%	
Sex					
Male	207	60.0%	25,724	52.7%	0.15
Female	138	40.0%	23,099	47.3%	
SARS-CoV-2 infection period					
March 2022 - May 2022	22	6.4%	8,546	17.5%	
June 2022 - September 2022	103	29.9%	25,736	52.7%	0.74
October 2022 - January 2023	220	63.8%	14,541	29.8%	
Symptomatic presentation	122	35.4%	26,787	54.9%	0.40
Pre-existing conditions					
Asthma	0	0.0%	3	0.0%	NA
Cancer	0	0.0%	1	0.0%	NA
Cardiac disease	0	0.0%	1	0.0%	NA
Lung disease	6	1.7%	42	0.1%	0.17
Mental disease	0	0.0%	3	0.0%	NA
Neurologic disease	0	0.0%	5	0.0%	NA
Obesity	1	0.3%	0	0.0%	NA
Diabetes mellitus	0	0.0%	3	0.0%	NA
Disabilities	0	0.0%	3	0.0%	NA
ADHD	0	0.0%	1	0.0%	NA
Autism	0	0.0%	0	0.0%	NA
Immunocompromised	2	0.6%	20	0.0%	0.10
Healthcare utilization	7	2.0%	2,645	5.4%	0.18
COVID-19 vaccination status*					
Not fully vaccinated	30	8.7%	12,418	25.4%	
Fully vaccinated but not boosted	76	22.0%	16,985	34.8%	0.65
Boosted	239	69.3%	19,420	39.8%	

Notes: *ADHD* attention deficit hyperactivity disorder, *SD* standard deviation, *SMD* standardized mean difference, *NA* not applicable

* Fully vaccinated but not boosted patients were defined as those with two doses of BNT162b2 (Comirnaty) or COVID-19 Vaccine (Vero Cell), Inactivated (CoronaVac); boosted patients were defined as those with at least three doses of BNT162b2 (Comirnaty) or COVID-19 Vaccine (Vero Cell), Inactivated (CoronaVac).

Supplementary Table 4. Cause of hospitalization among nirmatrelvir/ritonavir users and controls after cloning and censoring

Cause of hospitalization	Nirmatrelvir/ritonavir (N=211)		Controls (N=332)	
COVID-19	200	94.8%	215	64.8%
Infectious and Parasitic Diseases	1	0.5%	5	1.5%
Neoplasms	1	0.5%	5	1.5%
Endocrine, Nutritional and Metabolic Diseases, and Immunity Disorders	0	0.0%	3	0.9%
Diseases of the Blood and Blood-forming Organs	1	0.5%	1	0.3%
Mental Disorders	1	0.5%	16	4.8%
Diseases of the Nervous System and Sense Organs	0	0.0%	8	2.4%
Diseases of the Circulatory System	0	0.0%	1	0.3%
Diseases of the Respiratory System	0	0.0%	4	1.2%
Diseases of the Digestive System	1	0.5%	4	1.2%
Diseases of the Genitourinary System	0	0.0%	2	0.6%
Complications of Pregnancy, Childbirth, and the Puerperium	0	0.0%	0	0.0%
Diseases of the Skin and Subcutaneous Tissue	0	0.0%	3	0.9%
Diseases of the Musculoskeletal System and Connective Tissue	1	0.5%	5	1.5%
Congenital Anomalies	0	0.0%	1	0.3%
Certain Conditions originating in the Perinatal Period	0	0.0%	0	0.0%
Symptoms, Signs and Ill-defined Conditions	2	0.9%	22	6.6%
Injury and Poisoning	1	0.5%	31	9.3%
Supplementary Classification of Factors influencing Health Status and Contact with Health Services	2	0.9%	6	1.8%

Notes: Among 211 hospitalized nirmatrelvir/ritonavir users after cloning, 204 and 7 were admitted during and after the grace period, respectively. Among 332 hospitalized controls after cloning, 207 and 125 were admitted during and after the grace period, respectively.

Supplementary Table 5. Sensitivity analyses on the 28-day all-cause hospitalization outcome by (1) truncating the IPCW at the 1st and 99th percentiles to minimize the impact of extreme weights on the results; and (2) extending the follow-up duration till the end of observational period

	Cumulative incidence		Absolute risk reduction [a]	95% CI [b]	Relative risk [a]	95% CI [b]
	Nirmatrelvir/ Ritonavir (N=49,378)	Control (N=49,378)				
Truncating the inverse probability of censoring weights at the 1st and 99th percentiles	0.45%	0.68%	0.22%	(0.17%, 0.29%)	0.67	(0.58, 0.74)
Extending the follow-up duration till the end of observational period	0.45%	0.69%	0.24%	(0.20%, 0.32%)	0.65	(0.55, 0.70)

Notes: *CI* confidence interval

[a] Absolute risk reduction >0 (or <0) and relative risk <1 (or >1) indicate nirmatrelvir/ritonavir users had lower (higher) risk of the designated outcome compared to controls.

[b] The 95% CIs were calculated using 500 bootstrap replicates.

Supplementary Table 6. Cumulative incidences of secondary study outcomes at 28 days in nirmatrelvir/ritonavir and control groups

	Cumulative incidence		Absolute risk reduction [a]	95% CI [b]	Relative risk [a]	95% CI [b]
	Nirmatrelvir/ ritonavir	Control				
All-cause mortality	0.00%	0.00%	NA	NA	NA	NA
COVID-19-specific hospitalization	0.40%	0.44%	0.04%	(0.02%, 0.06%)	0.91	(0.85, 0.94)
In-hospital disease progression	0.00%	0.02%	0.01%	(0.00%, 0.03%)	0.22	(0.00, 0.54)
In-hospital death	0.00%	0.00%	NA	NA	NA	NA
Invasive mechanical ventilation	0.00%	0.00%	NA	NA	NA	NA
Intensive care unit admission	0.00%	0.01%	0.01%	(0.00%, 0.02%)	0.29	(0.00, 0.67)
Multisystem inflammatory syndrome in children (MIS-C)	0.00%	0.00%	NA	NA	NA	NA
Acute liver injury	0.00%	0.01%	0.01%	(0.00%, 0.02%)	0.33	(0.00, 0.75)
Acute renal failure	0.01%	0.02%	0.02%	(0.01%, 0.03%)	0.25	(0.00, 0.54)
Acute respiratory distress syndrome	0.01%	0.02%	0.00%	(0.00%, 0.01%)	0.74	(0.34, 1.00)

Notes: *CI* confidence interval, *NA* not applicable

[a] Absolute risk reduction >0 (or <0) and relative risk <1 (or >1) indicate nirmatrelvir/ritonavir users had lower (higher) risk of the designated outcome compared to controls.

[b] The 95% CIs were calculated using 500 bootstrap replicates.

Supplementary References

1 Hernan, M. A., Brumback, B. & Robins, J. M. Marginal structural models to estimate the causal effect of zidovudine on the survival of HIV-positive men. *Epidemiology* **11**, 561-570 (2000). https://doi.org/10.1097/00001648-200009000-00012

2 Emilsson, L. *et al.* Examining Bias in Studies of Statin Treatment and Survival in Patients With Cancer. *JAMA Oncol* **4**, 63-70 (2018). https://doi.org/10.1001/jamaoncol.2017.2752

3 Yousaf, A. R. *et al.* Reported cases of multisystem inflammatory syndrome in children aged 12-20 years in the USA who received a COVID-19 vaccine, December, 2020, through August, 2021: a surveillance investigation. *Lancet Child Adolesc Health* **6**, 303-312 (2022). https://doi.org/10.1016/S2352-4642(22)00028-1

4 Devarbhavi, H. *et al.* Drug-induced liver injury: Asia Pacific Association of Study of Liver consensus guidelines. *Hepatol Int* **15**, 258-282 (2021). https://doi.org/10.1007/s12072-021-10144-3

5 Khwaja, A. KDIGO clinical practice guidelines for acute kidney injury. *Nephron Clin Pract* **120**, c179-184 (2012). https://doi.org/10.1159/000339789
